# Changes in the expression and function of the PDE5 pathway in the obstructed urinary bladder

**DOI:** 10.1111/jcmm.15926

**Published:** 2020-10-03

**Authors:** Weixiang He, Han Xiang, Daoquan Liu, Jianmin Liu, Mingzhou Li, Qian Wang, Qiaofeng Qian, Yan Li, Xun Fu, Ping Chen, Yuming Guo, Guang Zeng, Zhonghua Wu, Daxing Zhan, Xinghuan Wang, Michael E. DiSanto, Xinhua Zhang

**Affiliations:** ^1^ Department of Urology Zhongnan Hospital of Wuhan University Wuhan China; ^2^ Department of Urology The Second Affiliated Hospital of Soochow University Suzhou China; ^3^ Department of Anesthesiology Zhongnan Hospital of Wuhan University Wuhan China; ^4^ Department of Surgery and Biomedical Sciences Cooper Medical School of Rowan University Camden NJ USA

**Keywords:** benign prostate hyperplasia, lower urinary tract symptoms, partial bladder outlet obstruction, phosphodiesterase type 5

## Abstract

Our study aims to explore changes in bladder contractility and the phosphodiesterase type 5 (PDE5) signalling pathway in response to partial bladder outlet obstruction (PBOO). A surgically induced male rat PBOO model and human obstructed bladder tissues were used. Histological changes were examined by H&E and Masson's trichrome staining. Bladder strip contractility was measured via organ bath. The expressions of nitric oxide synthase (NOS) isoforms, PDE5, muscarinic cholinergic receptor (CHRM) isoforms and PDE4 isoforms in bladder were detected by RT‐PCR and Western blotting. The immunolocalization of the PDE5 protein and its functional activity were also determined. PBOO bladder tissue exhibited significant SM hypertrophy and elevated responsiveness to KCl depolarization and the muscarinic receptor agonist carbachol. NOS isoforms, PDE5, CHRM2, CHRM3 and PDE4A were up‐regulated in obstructed bladder tissue, whereas no change in PDE4B and PDE4D isoform expression was observed. With regard to PDE5, it was expressed in the SM bundles of bladder. Interestingly, obstructed bladder exhibited less relaxation responsiveness to sodium nitroprusside (SNP), but an exaggerated PDE5 inhibition effect. The up‐regulation of PDE5 could contribute to the lack of effect on Q_max_ for benign prostatic hyperplasia/lower urinary tract symptom (BPH/LUTS) patients treated with PDE5 inhibitors. Moreover, PDE5 (with presence of NO) and PDE4 may serve as new therapeutic targets for bladder diseases such as BPH‐induced LUTS and overactive bladder (OAB).

## 1 INTRODUCTION

Bladder outlet obstruction (BOO) resulting from benign prostate hyperplasia (BPH) is a common problem among elderly males, with a high incidence of lower urinary tract symptoms (LUTS), such as incomplete bladder emptying, weak urine stream, urinary frequency, urgency, urge incontinence and nocturia.^1^ However, the mechanisms of LUTS induced by BOO remain controversial. LUTS can be caused not only by prostatic obstruction, but also by bladder dysfunction,^2^ such as detrusor overactivity (DO) and overactive bladder (OAB). DO is a urodynamic observation characterized by involuntary detrusor contractions during the filling phase which may be spontaneous or provoked.^3^ OAB syndrome is characterized by urinary urgency, daytime frequency and nocturia with or without urgency urinary incontinence.^4^

Currently, oral drug treatment for male BPH/LUTS recommended by guidelines^5^, ^6^ includes α1‐adrenoceptor antagonists (α1‐blockers), 5α‐reductase inhibitors (5‐ARIs), muscarinic receptor antagonists (MRAs) and a ‘newly emerging treatment’ that utilizes phosphodiesterase type 5 inhibitors (PDE5is). PDE5is are the first‐line treatment for erectile dysfunction (ED) with sildenafil, vardenafil and tadalafil commonly used. Since the first clinical trial conducted by Sariam^7^ in 2002 employing sildenafil for treating BPH/LUTS/ED patients, the effect of PDE5is on BPH/LUTS has been extensively explored. However, only tadalafil (5 mg once daily), a long‐acting PDE5is, has been licensed for moderate‐to‐severe LUTS with or without ED.^5^, ^6^ The majority of randomized controlled trials (RCTs) have demonstrated that PDE5is could reduce International Prostate Symptom Score (IPSS), relieve storage and voiding LUTS and improve the quality of life (QoL) without significantly altering the maximal urine flow rate (Q_max_).^6^ Consistently, our previous network meta‐analysis study^8^ showed that PDE5is could reduce the total IPSS score, storage IPSS and voiding IPSS, and rank higher for the improvement of LUTS/BPH when compared with other therapies.^9^ Again, no significant effect on Q_max_ was found for PDE5is.^8^ Gacci^10^ postulated that PDE5is not only relaxed the prostate SM and bladder neck to reduce the IPSS score, but also relaxed detrusor SM, which may counteract the relaxation effect on the bladder outlet and lead to no improvement on Q_max_.

Different from α1‐blockers treating BPH/LUTS through lessening the resistance from the urethra muscle, the potential mechanism of PDE5is in treating BPH/LUTS may be multifaceted. Besides the expression and functional activity of PDE5 in bladder, prostate and urethra,^11^, ^12^ blood vessels that supply the prostate and bladder also express high level of active PDE5.^13^ In general, PDE5is could relax SM via acting on the nitric oxide/cyclic guanosine monophosphate (NO/cGMP) signalling pathway, which was believed to play a central role in the pathophysiology of LUTS. Specifically, NO produced by nitric oxide synthase (NOS) enters SM cells then via guanylate cyclase increases the production of cGMP, a kind of second messenger which can relax SM cells. PDE5 can degrade cGMP and stop the relaxation of SM. On the other hand, its inhibitors could block PDE5 activity which results in SM relaxation. In addition, the plausible mechanism of PDE5is in treating BPH/LUTS may be summarized as follows: (a) relief of SM tone in prostate and bladder^14^, ^15^; (b) relaxation of blood vessels so as to increase LUT perfusion and oxygenation ^16^; (c) decreasing the activity of afferent nerves in the LUT to modulate the micturition reflex ^17^, ^18^, ^19^; (d) anti‐proliferation effect on bladder^20^ and prostate ^12^; and (e) blunting of intraprostatic inflammation^21^ and an anti‐fibrotic effect on bladder.^22^ However, bladder PDE5 has been less studied. The aim of current study was to explore whether expression and functional activity of PDE5 in the bladder wall were altered in response to partial bladder outlet obstruction (PBOO) in rats and humans. Also, some other molecules related to bladder SM contraction and relaxation pathways were evaluated.

## 2 MATERIALS AND METHODS

### 2.1 Rat partial bladder outlet obstruction model

Thirty specific‐pathogen‐free (SPF) grade male Sprague Dawley (SD) rats bought from Beijing Vital River Laboratory Animal Technology Co., Ltd. (Beijing, China) were used and randomly divided into a PBOO group and sham group. All animals were kept on the normal chow with a 12 hour day/night light cycle for more than one week to adapt to the new environment. As previously reported,^23^ rats from the PBOO group were anaesthetized with pentobarbital (35 mg/kg) via an intraperitoneal (ip) injection. A 2‐cm midline vertical incision was made from the penoscrotal junction to the midscrotum to gain access to the bulbous urethra. The urethra was then isolated from the cavernosum bodies. A sterile metal bar (19‐gauge needle) with a 1.06 mm diameter was placed on the bulbous urethral surface, and a 3‐0 polypropylene suture was used to place a tie around both the bulbous urethra and the bar. As soon as the suture was secured, the bar was removed, leaving the bulbous urethra partial obstructed. A 4‐0 silk suture was used to reapproximate the muscle layer, and a 4‐0 nylon suture was used to close the skin. Sham surgery was performed the same as described above except that the urethral ties were not placed. All animals were kept for 2 weeks on normal chow with a 12 hour day/night light cycle. At the end of 2 weeks, rats were anaesthetized by isoflurane (in 100% oxygen, 5% for induction and 1.5% for maintenance) at a flow rate of 1 L/min and killed by cervical dislocation. Their bladder, prostate and seminal vesicles were quickly obtained. Animal experiments were conducted at the Animal Center of Zhongnan Hospital of Wuhan University, and all animal protocols were approved by the Medical Ethics Committee for Experimental animals of Zhongnan Hospital of Wuhan University. The pain and suffering of experimental rats were kept to a minimum.

### 2.2 Human bladder tissue acquisition

Human obstructed bladder tissue was obtained from ten male patients that needed to undergo radical cystectomy and whose IPSS score was above 19 points and pathological results demonstrated BPH. All samples were identified by two separate pathologists and showed no tumour infiltration. Normal bladder tissue was obtained from ten male brain‐dead men undergoing donation in the transplant centre of Zhongnan Hospital, who were less than 40 years old, and pathological examination showed no hyperplasia (human data are listed in Table 1). All human samples were obtained after the approval of the Hospital Committee for Investigation in Humans and after receiving written informed consent from all patients or their relatives. All human studies were conducted in accordance with the principles of the Declaration of Helsinki.

**Table 1:** Clinical information of BOO patients and brain‐dead men

	BOO (n = 10)	Control (n = 10)	*P*‐value
Age (years)	70.38 ± 6.05	31.54 ± 6.75	<0.0001
BMI (kg/m^2^)	22.75 ± 3.14	21.39 ± 2.98	0.5856
IPSS	23.75 ± 3.84	NA	NA
Prostate volume	55.28 ± 17.02	NA	NA

Note

Data are expressed as mean ± SD.

NA, not applicable.

### 2.3 H&E Staining and Masson's Trichrome Staining

Rat and human bladder tissues fixed in 10% neutral buffered formalin for 48 hour were processed routinely for paraffin embedding. The paraffin‐embedded tissue sections (4 μm) were stained with haematoxylin and eosin using standard techniques. The paraffin sections were deparaffinized in xylene, followed by graded alcohols. Masson's composite staining solution (Fuzhou Maxim Biotech Co., Ltd., Fuzhou, China) was added dropwise for 10 minutes. The sections were subsequently washed with distilled water, differentiated in phosphomolybdic‐phosphotungstic acid solution for 10 minutes and incubated with blue staining solution for 5‐10 minutes. Next, the sections were rinsed briefly in distilled water and differentiated in 1% acetic solution for 2 minutes. After being dehydrated quickly through 95% alcohol and absolute alcohol, the sections were cemented using neutral gum for observation. Using this procedure, bladder SM cells were stained red and collagen fibres were stained blue. In each sample, we analysed three areas under magnification (×100). The choice of three fields was randomized without specific areas of a demarcated slide. The area percentage of SM and collagen fibres were quantitated with Image‐Pro Plus 6.0, respectively. Moreover, in each sample, three random areas under magnification (×25) were chosen and the diameter and number of detrusor SM cells in these micrographs were measured.

### 2.4 In vitro organ bath studies

As previously described,^23^ bladder strips containing urothelium with identical length were mounted longitudinally in a 10 mL organ bath (Multi‐Myograph Model 810MS; Danish Myo Technology; Aarhus, Denmark). The dimension of bladder strips was approximately 1 × 0.5 × 0.5 cm and 1 × 1 × 0.15 cm, for human and rat respectively. The myograph was connected in line to a PowerLab 4/30 Data Acquisition System (ADInstruments; Colorado Springs, CO, USA) and in turn to a Dual‐Core Processor Pentium computer for real‐time monitoring of physiological force. The bladder SM strips were equilibrated at least 1 hour in Krebs‐Henseleit (Krebs) buffer^23^ at 37°C with continuous bubbling of 95% O_2_ and 5% CO_2_. The buffer had the following mmol/L composition: NaCl 110, KCl 4.8, CaCl_2_ 2.5, MgSO_4_ 1.2, KH_2_PO_4_ 1.2, NaHCO_3_ 25 and dextrose 11, and it was changed every 15 minutes. Strips were continuously adjusted to 15‐20 mN resting tension. After equilibration, bladder strips were contracted with 60 mmol/L KCl and, after washing with buffer to baseline, cumulative concentrations (10^−8^‐10^−4^) of carbachol (CC). The maximum force of KCl depolarization was taken as 100%, and the force generated by CC was normalized to a percentage of this value. After the contraction experiment completed, the strips were washed several times using buffer and the tension was reduced to the baseline. Next, strips were pre‐contracted with 1 μmol/L carbachol (a dose that induces about 50% maximal contraction) and allow to reach stable tension and then the relaxant effects of increasing doses of sodium nitroprusside (SNP) with or without 100 nmol/L vardenafil were evaluated. The concentration of 100 nmol/L was chosen because it reflects the C_max_ of vardenafil reached in clinical studies with 10 mg dosing to humans.^24^ The effects of SNP were also compared with vehicles (saline). Moreover, the relaxant effects of increasing doses of vardenafil were also evaluated. Finally, for the concentration‐effect curve, EC50s and a maximal effect value were determined using the four parameter logistic model by GraphPad Prism 7.0.

### 2.5 Total RNA extraction and real‐time RT‐PCR

Total RNA was isolated from the tissues using TaKaRa MiniBEST Universal RNA Extraction Kit (Takara Bio. Inc, Otsu, Shiga, Japan) according to the manufacturer's protocol. 100 ng of RNA was added in the one‐step real‐time RT‐PCR reaction system (Takara Bio. Inc). The whole system was amplified in a 96‐well plate in a 25 μL reaction volume with all samples run in triplicate, using a CFX96 Touch Real‐Time PCR Detection System (Bio‐Rad, USA). The experimental protocol utilized was first reverse transcription (42°C for 5 minutes, 95°C for 10 seconds), followed by an amplification program repeated for 40 cycles (95°C for 5 seconds, then 60°C for 30 seconds), using SYBR Green measurement. For human and rat urinary bladder tissue, the following targets were amplified: PDE5, NOS isoforms (eNOS and nNOS), CHRM isoforms (CHRM2 and CHRM3) and PDE4 isoforms (PDE4A, PDE4B and PDE4D). Primer sequences are shown in Tables 2&3. For relative quantification, gene expression was normalized to expression of β‐actin housekeeping gene and compared by 2^−ΔΔCT^ method.

**Table 2:** Primer sequences used to amplify target genes in rat by real‐time RT‐PCR

Target gene	Primer sequence
*PDE5A*	
Forward	5′‐TTGGAGAGCCCTTGAACATCA‐3′
Reverse	5′‐GTAGCCTGTAATTTGGTCAACTTCTG‐3′
*eNOS*	
Forward	5′‐GCCTGAGCAGCACAAGAGTTAC‐3′
Reverse	5′‐CCAGCCCAAACACACAGAACC‐3′
*nNOS*	
Forward	5′‐GGCAAACATGACTTCCGAGTGT‐3′
Reverse	5′‐CCCCAAGGTAGAGCCATCTG‐3′
*CHRM2*	
Forward	5′‐GCAATGCCTCCGTTATGAAT‐3′
Reverse	5′‐GTGGTCCGCTTAACTGGGTA‐3′
*CHRM3*	
Forward	5′‐TCATCCAGGAGGAAGTACGG‐3′
Reverse	5′‐GCTGTGGTCTTGGTCCATCT‐3′
*PDE4A*	
Forward	5′‐GTGGAGAAGTCTCAGGTGGG‐3′
Reverse	5′‐TGGAACTTGTCAGGCAGGG‐3′
*PDE4B*	
Forward	5′‐TAGAAGATAACAGGAACTGG‐3′
Reverse	5′‐GCAATGTCTATGTCAGTCTC‐3′
*PDE4D*	
Forward	5′‐GGATAATGGAGGAGTTCTTCC‐3′
Reverse	5′‐CGATTGTCCTCCAAAGTGTCC‐3′
*β‐actin*	
Forward	5′‐ACCAACTGGGACGATATGGAGAAGA‐3′
Reverse	5′‐TACGACCAGAGGCATACAGGGACAA‐3′

**Table 3:** Primer sequences used to amplify target genes in human by real‐time RT‐PCR

Target gene	Primer sequence
*PDE5A*	
Forward	5′‐CCTCCATCACGAGAGTCATTTCAG‐3′
Reverse	5′‐CCACCTTGACCATCTCATGACTTTA‐3′
*eNOS*	
Forward	5′‐CCAGCTAGCCAAAGTCACCAT‐3′
Reverse	5′‐GTCTCGGAGCCATACAGGATT‐3′
*nNOS*	
Forward	5′‐GGAAGCCCCATAATTGTTCT‐3′
Reverse	5′‐ATTGGATGGGTTCATGTCAC‐3′
*CHRM2*	
Forward	5′‐TCACAAAACCTCTGACCTACCC‐3′
Reverse	5′‐TCCACAGTTCTCACCCCTACAA‐3′
*CHRM3*	
Forward	5′‐CACCATCCTCAACTCCACCAAGT‐3′
Reverse	5′‐GGAAAACTGCCTCCATCGTC‐3′
*PDE4A*	
Forward	5′‐GCCCCCTGTACCACACTTAC‐3′
Reverse	5′‐TGTCACCATCGTGTCCACAG‐3′
*PDE4B*	
Forward	5′‐ATTGTGGGAGACATGGGCAG‐3′
Reverse	5′‐AAAGCGTCTTTGTGCTGCTG‐3′
*PDE4D*	
Forward	5′‐TCAGAGTGGTAAATTGTGTGT‐3′
Reverse	5′‐GGCAGAATCAACCCATGCTT‐3′
*β‐actin*	
Forward	5′‐TGACGTGGACATCCGCAAAG‐3′
Reverse	5′‐CTGGAAGGTGGACAGCGAGG‐3′

### 2.6 SDS‐PAGE and Western blotting analysis

As previously described, proteins were extracted from tissues using RIPA (radioimmunoprecipitation assay) lysis buffer (Sigma‐Aldrich, St Louis, MO, USA) (containing 1 × PBS, 1% IGEPAL CA‐630, 0.5% sodium deoxycholate, 0.1% SDS, 10 mmol/L EDTA) with freshly added phenylmethanesulphonyl fluoride (PMSF; Sigma‐Aldrich) and sodium orthovanadate (Sigma‐Aldrich). Then, a BCA protein assay kit (Beyotime, Wuhan, China) was used to determine the protein concentration. An aliquot of 100μg of each sample was electrophoresed on a 7.5% or 12.5% sodium dodecyl sulphate‐polyacrylamide (SDS‐PAGE) gel (Wuhan Boster Biological Technology Ltd, Wuhan, China) and transferred to polyvinylidene fluoride (PVDF) membrane (Millipore, Billerica, MA, USA) using a Bio‐Rad wet transfer system. The membrane was blocked for 2 hours at room temperature with Tris‐buffered saline with 0.1% [v/v] Tween (TBST) containing 5% [w/v] non‐fat dry milk solution. Membranes were cut into proper size according to the molecular weight of target proteins indicated by the manufacturer's instructions of antibodies and then incubated overnight with corresponding protein primary antibodies. (Information of primary antibodies was listed in Table 4) After washing, the membranes were incubated with secondary antibody at room temperature for 2 hours. Detection of reaction antigen was performed with an enhanced chemiluminescence (ECL) kit (Thermo Fisher Scientific, Waltham, MA, USA). The bands were quantified by reflectance scanning of gel photographs obtained with a BioDoc XRS + camera using Bio‐Rad Molecular Imager^®^ ChemiDoc™ XRS + System and Quantity One^®^ SW 1‐D Analysis software (Bio‐Rad). Glyceraldehyde 3‐phosphate dehydrogenase (GAPDH) was used as the loading control to normalize the protein expression.

**Table 4:** List of primary antibodies

Protein target	Name of antibody	Manufacturer and catalog	Species raised in; monoclonal or polyclonal	Dilution used
PDE5	Anti‐PDE5A/PDE5 Antibody	Abcam, ab64179	Rabbit polyclonal	1:1000 (WB) 1:100 (IF)
eNOS	NOS3 Antibody (B‐5)	Santa Cruz, sc‐136977	Mouse monoclonal	1:1000 (WB)
nNOS	NOS1 Antibody (A‐11)	Santa Cruz, sc‐5302	Mouse monoclonal	1:1000 (WB)
CHRM2	Anti‐CHRM2 Antibody	Abcam, ab109226	Rabbit monoclonal	1:1000 (WB)
CHRM3	CHRM3 Antibody	ABclonal, A1602	Rabbit polyclonal	1:1000 (WB)
PDE4A	Anti‐PDE4A Antibody	Abcam, ab14607	Rabbit polyclonal	1:1000 (WB)
PDE4B	Anti‐PDE4B Antibody	Abcam, ab14611	Rabbit polyclonal	1:1000 (WB)
PDE4D	PDE4D Antibody	ABclonal, A1659	Rabbit polyclonal	1:1000 (WB)
GAPDH	GAPDH Antibody	ABclonal, AC001	Rabbit polyclonal	1:1000 (WB)
α‐SMA	Smooth Muscle Actin Antibody (B4)	Santa Cruz, sc‐53142	Mouse monoclonal	1:100 (IF)

### 2.7 Immunofluorescence microscopy

Human and rat urinary bladders were embedded in Tissue‐Tec OCT compound (Sakura Finetek Japan, Tokyo, Japan) and snap‐frozen. Then, the tissue was sectioned in 10‐μm‐thick slices, thawed and then mounted onto glass slides using a cryostat (Leica CM 1850, Wetzlar, Germany). The sections were then air‐dried and fixed for 10 minutes in ice‐cold acetone. Tissue Autofluorescence Quenching Agent (#G1221, Servicebio, Wuhan, China) was used according to the manufacturer's instructions. Then, slides were washed in PBS and next incubated for 2 hour in a mixture of PBS supplemented with 0.2% [v/v] Triton X‐100 and 0.1% [w/v] bovine serum albumin, followed by incubation overnight with the primary antibody α‐SMA (mouse polyclonal to α‐smooth muscle actin, 1:100) and antibody mixture of the PDE5 antibody (rabbit polyclonal to PDE5A, 1:100). The secondary antibodies employed to visualize the localization of the two primary antibodies (Jackson ImmunoResearch Inc West Grove, PA, USA) were labelled with Cy2‐conjugated antimouse IgG (1:200) and Cy3‐conjugated anti‐rabbit IgG (1:1000). DAPI was used for staining the nucleus. Negative controls were performed for all samples by omitting the primary antibodies. Human and rat lung tissues were used as a positive control for PDE5A staining, respectively. Visualization was done with a laser microscope (Olympus, Tokyo, Japan). Graphs were obtained from laser scanning at 405 nm, 488 nm and 561 nm wavelength of excitation laser line. Colocalization analysis was performed using the NIS‐Elements Viewer 3.20 (Nikon, Japan).

### 2.8 Statistical analysis

Results are expressed as mean ± SEM for n experiments. Statistical analysis used either the Student's t test with Excel software (two‐sample treatments compared) or ANOVA and Bonferroni's post‐tests with GraphPad Prism 7.0 (multiple means compared). *P* < 0.05 was considered significant.

## 3 RESULTS

### 3.1 Rat bladder weight is increased in PBOO model

**Figure 1:**
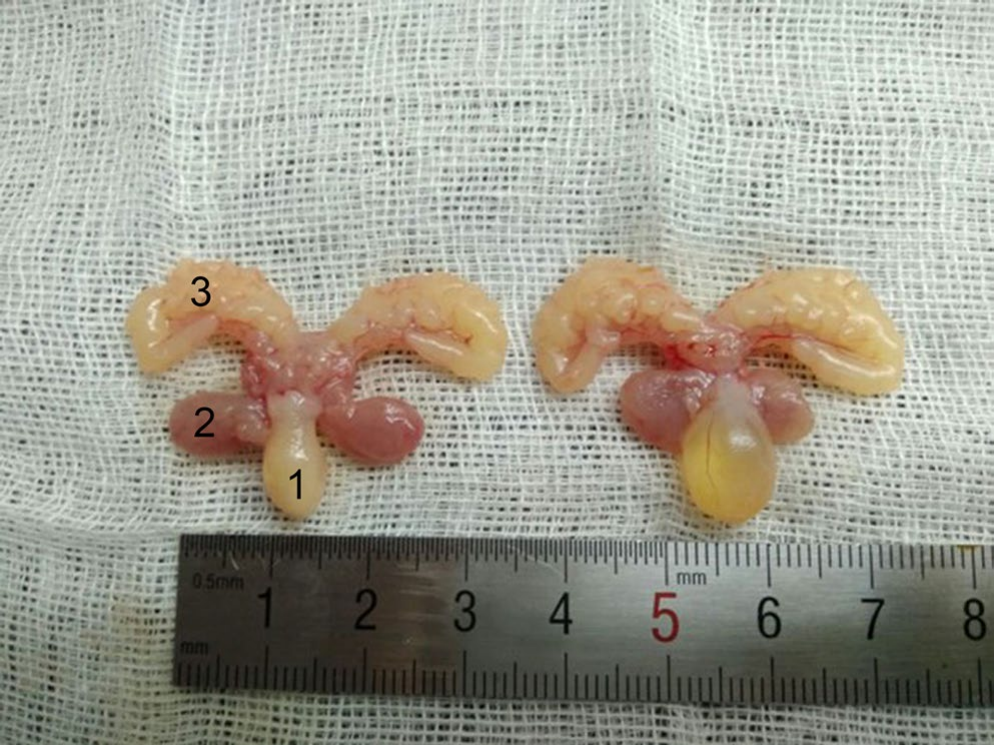
Photographs of urinary bladder, prostate and seminal vesicle from PBOO and sham rat. Photograph of a typical urinary bladder (1), prostate (2) and seminal vesicle (3) from sham (left) and PBOO rats (right)

In rats, enlarged bladder mass was observed in the PBOO group (Figure 1) with mean bladder weight significantly increased from 128.0 ± 29.7 mg to 263.0 ± 89.3 mg (*P* < 0.01) representing a 2.05‐fold increase. Since the bodyweight did not increase as much in the PBOO rats, the bladder weight‐to‐bodyweight ratio actually increased by 2.43‐fold (Table 5).

**Table 5:** Rat bodyweight and bladder weight

Group	Bodyweight (g)		Bladder weight (mg)	%Bladder weight/Bodyweight
	Initial	Final		
Sham (n = 10)	228.1 ± 11.7	329.9 ± 16.2	128.0 ± 29.7	0.039 ± 0.002
PBOO (n = 10)	226.7 ± 8.8	275.6 ± 19.7**	263.0 ± 89.3**	0.095 ± 0.008**

Note

Data are expressed as mean ± SD.

** *P* < 0.01 vs sham.

### 3.2 Histological and pathological changes in PBOO rat and human bladder

**Figure 2:**
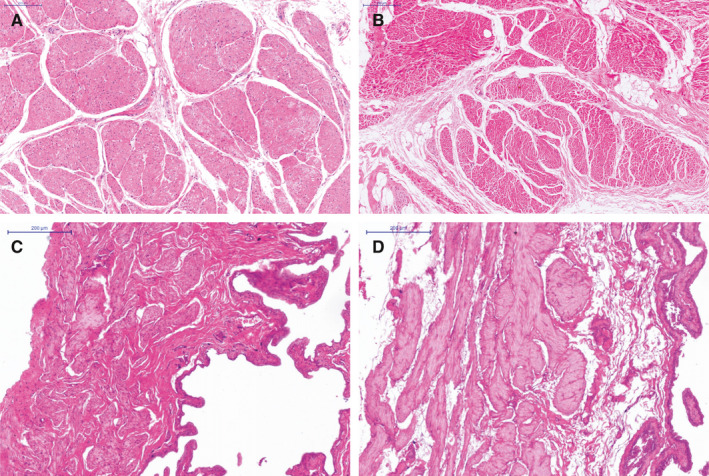
Haematoxylin and eosin (H&E) staining of human and rat urinary bladder. Representative sections of human urinary bladder from control (A) and PBOO group (B) and rat urinary bladder from sham (C) and PBOO (D) group. All sections were with magnification × 200

**Figure 3:**
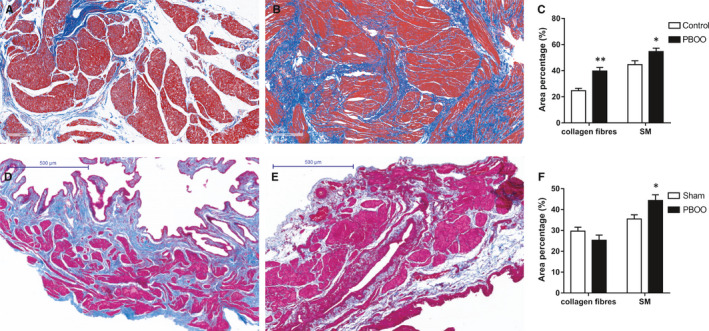
Masson's trichrome staining of human and rat urinary bladder and analysis. Masson's trichrome staining for control (A) and PBOO (B) human urinary bladder and sham (D) and PBOO (E) rat urinary bladder, respectively (magnification × 100). Urinary bladder smooth muscle (SM) cells were stained red and collagen fibres were stained blue. C, The bar graph for area percentage of different components (collagen fibres and SM) between control and PBOO groups (n = 10 different humans for each group). White column, control; black column, PBOO. F, The bar graph for area percentage of different components between sham and PBOO groups (n = 10 different rats for each group). White column, sham; black column, PBOO. Data were shown as mean ± SEM. *P < 0.05 vs sham

H&E staining showed that PBOO rat and human bladder exhibited SM hypertrophy, arrangement disorder and fibrous tissue invaded the SM bundles (Figure 2). Note that the rat bladder wall was observed obviously thickened in the PBOO group (Figure 2C,D). Meanwhile, Masson's trichrome staining (Figure 3) further showed the percentage of SM increased (*P* < 0.05) both in PBOO rat and human bladder, whereas the percentage of collagen fibres was only significantly increased in PBOO patients (*P* < 0.01). Moreover, both in humans and rats, diameter of SM cells from obstructed bladders increased, whereas the number of cells in a same size area showed no change between groups (Table S1).

### 3.3 In vitro contractility of bladder strips in response to KCl and carbachol are altered in obstructed bladder

**Figure 4:**
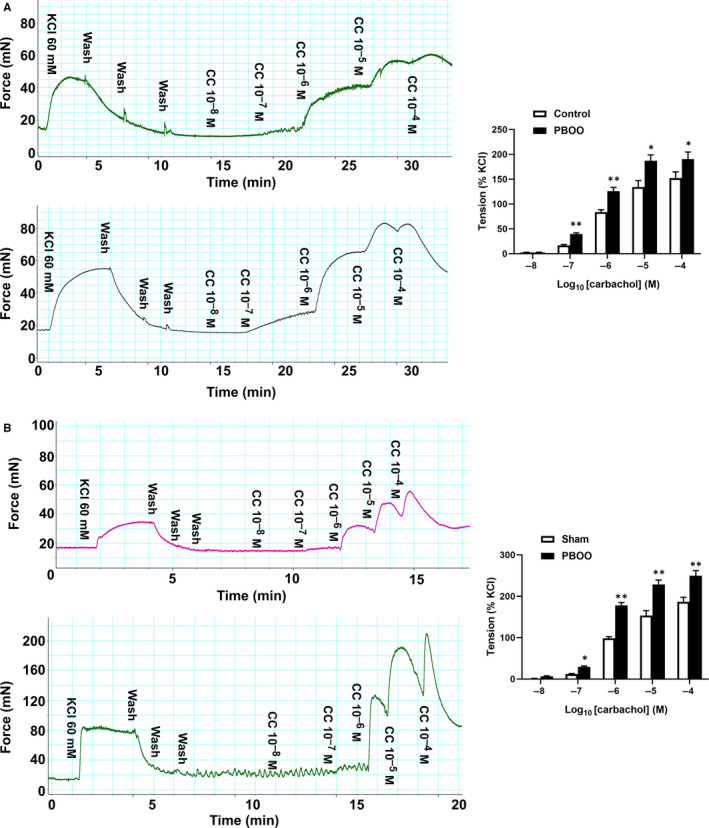
Contractility of urinary bladder strips and analysis. A, The left panels are force tracings of human urinary bladder SM contractions in response to KCl and carbachol (CC) from control (above) and PBOO patients (below). The x‐axis represents time (min), whereas the y‐axis represents force (mN). The right panels are summary graphs for contraction force of urinary bladder in response to cumulative doses (10^−8^‐10^−4^) of CC from control and PBOO patients (n = strips obtained from 10 different humans, one strip was used for each human). The maximum force of KCl depolarization was taken as 100% and the force generated by CC was normalized as a percentage of this value. Data are shown as mean ± SEM. *P < 0.05 vs control; **P < 0.01 vs control. B, The left panels are force tracings of rat urinary bladder SM contractions in response to KCl and carbachol (CC) from sham (above) and PBOO rats (below). The *x*‐axis represents time (min), whereas the *y*‐axis represents force (mN). The right panels are summary graphs for contraction force of urinary bladder in response to cumulative doses (10^−8^‐10^−4^) of CC from sham and PBOO group (n = strips obtained from 10 different rats, one strip was used for each rat). The maximum force of KCl depolarization was taken as 100% and the force generated by CC was normalized as a percentage of this value. Data are shown as mean ± SEM. *P < 0.05 vs sham; **P < 0.01 vs sham

As shown in Figure 4, in response to KCl depolarization and carbachol (CC), human and rat bladder strips produced significant force, and in a dose‐dependent manner for CC. The left panels of Figure 4A,B are representative tracings of human and rat bladder strips contraction in response to KCl and increasing doses (10^−8^‐10^−4^ mol/L) of CC. The maximum force of KCl depolarization was taken as 100%, and the force generated by CC was normalized as a percentage of this value. In human bladder, the EC50s of carbachol were 0.82 μmol/L and 0.46 μmol/L for control and PBOO groups, respectively. For rat bladder, the EC50s of carbachol were 0.92 μmol/L and 0.47 μmol/L for sham and PBOO groups, respectively. At 1 μmol/L, whether for human or rat, the tension of the bladder strips was near 50% of maximum contraction and this contraction was chosen for later experiments.

In addition, different from human bladder, rat PBOO bladder exhibited an increased shortening velocity as reflected by an approximately twofold faster time to 50% maximum contraction in response to both KCl and carbachol (12.43 ± 3.27 vs 23.12 ± 5.35 S and 8.24 ± 3.41 vs 15.72 ± 4.51 S, respectively) and much worse maintenance of force.

### 3.4 In vitro relaxation effect of SNP is attenuated and restored by vardenafil in bladder strips from PBOO human and rat

**Figure 5:**
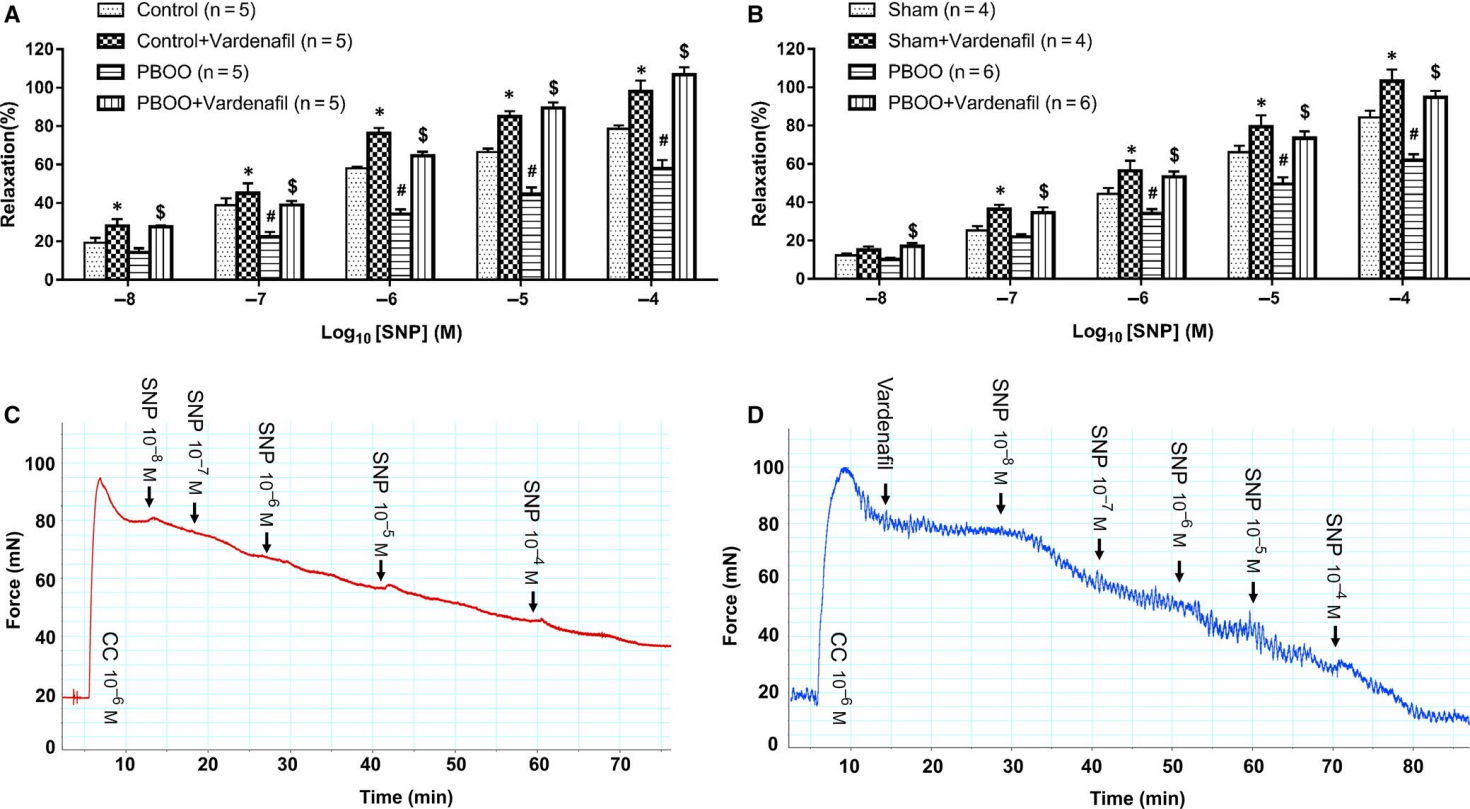
Relaxation response induced by SNP, with or without vardenafil. Bladder strips were pre‐contracted with 1 μΜ carbachol and allowed to reach a stable tension and then relaxed by increasing doses (10^−8^‐10^−4^ mol/L) of SNP with or without 100nM vardenafil pre‐incubation. The maximal response to carbachol was taken as 100%, whereas the relaxant effect of SNP with or without vardenafil was evaluated as a percentage of this response. A, Relaxation response of human bladder strips (n = 5 bladder strips from different humans). *P < 0.05 vs control at the same SNP dose; ^#^ *P* < 0.05 vs control at the same SNP dose; ^$^ *P* < 0.05 vs PBOO at the same SNP dose. B, Relaxation response of rat bladder strips (n = 4 or 6 bladder strips from different animals). *P < 0.05 vs sham at the same SNP dose; ^#^ *P* < 0.05 vs sham at the same SNP dose; ^$^ *P* < 0.05 vs PBOO at the same SNP dose. Values are expressed as mean ± SEM. C, Typical tracing of rat PBOO bladder strips relaxation in response to increasing doses (10^−8^‐10^−4^ mol/L) of SNP. D, Typical tracing of rat PBOO bladder strips relaxation in response to increasing doses (10^−8^‐10^−4^ mol/L) of SNP with pre‐incubation with vardenafil. The x‐axis represents time (min), whereas the y‐axis represents force (mN)

As shown in Figure 5, we explored the in vitro relaxation effect of SNP on bladder strips with or without vardenafil. To eliminate the effect of PBOO on NO formation, we studied the relaxation response by increasing doses SNP (10^−8^‐10^−4^ mol/L). Increasing concentrations of SNP induced a dose‐dependent relaxation response of bladder strips from both human and rat. In addition, vardenafil enhanced SNP‐induced bladder relaxation, at almost all the dose of SNP tested (10^−8^‐10^−4^ mol/L). Meanwhile, we also evaluated the effect of SNP when using vehicle as control (Figure S1). For both human and rat bladder strips pre‐contracted with CC, SNP exhibited a significant relaxant effect compared to vehicle controls.

Moreover, we explored the effect of vardenafil alone on pre‐contracted bladder strips. As shown in Figure S2, vardenafil caused concentration‐dependent relaxation for preparations. In CC pre‐contracted strips (in Figure S2A,B), this relaxant effect was particularly marked at higher concentrations, whereas vardenafil only marginally relaxed preparations at lower concentration. The ‐logEC50s were 4.81 and 4.77 for human control and PBOO patient bladder (Figure S2A), and 4.37 and 4.25 for rat sham and PBOO bladder (Figure S2B), respectively. Meanwhile, experiments using KCl pre‐contracted bladder strips showed similar effects of vardenafil (in Figure S2C,D). The −logEC50 were 4.79 and 4.50 for human control and PBOO bladder (Figure S2C), 4.83 and 4.77 for rat sham and PBOO bladder (Figure S2D), respectively.

### 3.5 Co‐immunolocalization of α‐SMA and PDE5 in human and rat bladder

**Figure 6:**
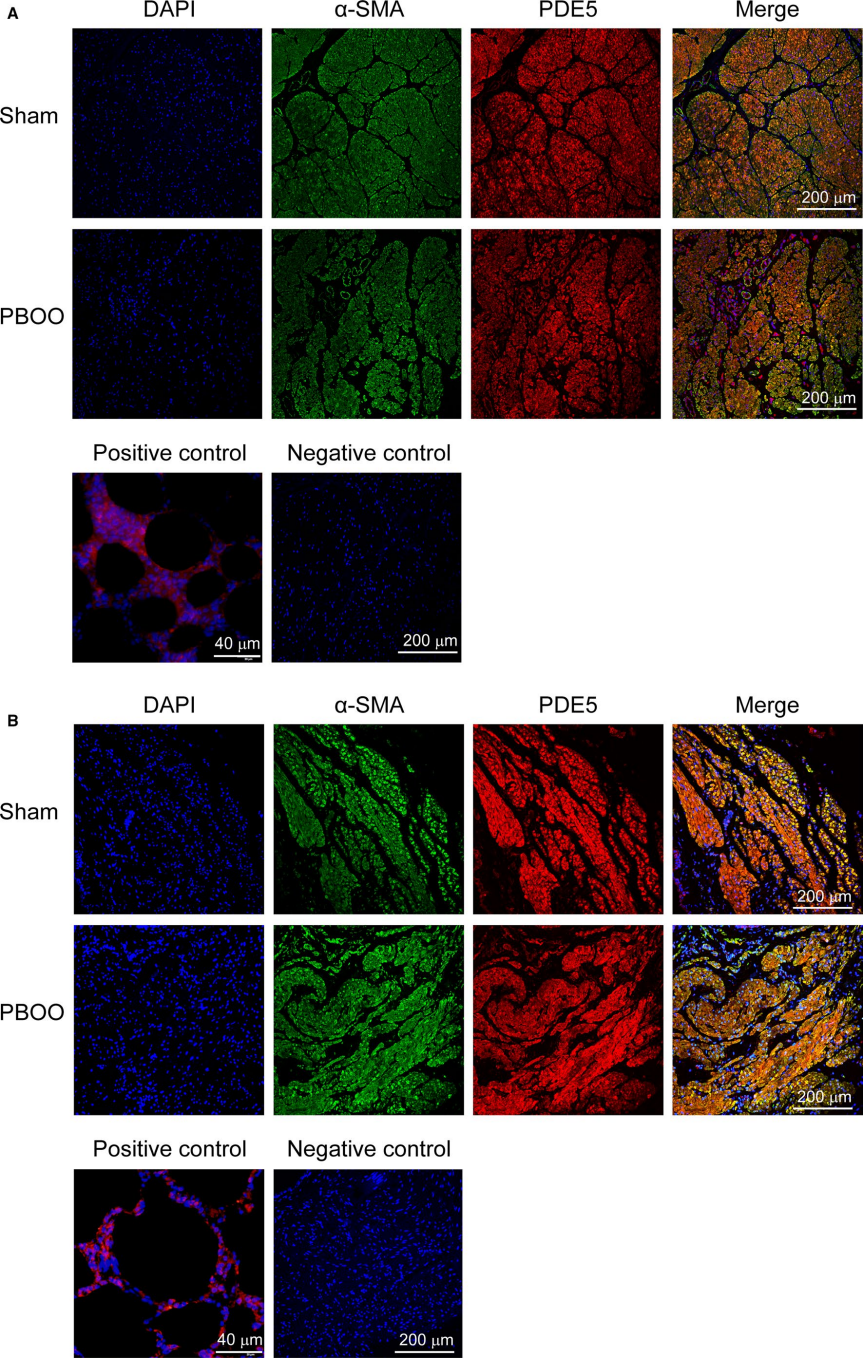
Immunolocalization of PDE5 in human and rat urinary bladder. A, The top and middle panels are immunolocalization of PDE5 in human urinary bladder. Representative double immunofluorescence staining for control (top) and PBOO patients (middle) bladder was conducted. DAPI (blue fluorescence), α‐SMA (green fluorescence), PDE5 (red fluorescence) and merged images are shown from left to right (magnification × 200). The bottom panels were positive control for PDE5 using human lung tissue (magnification × 400) and negative control by omitting the primary antibody (magnification × 200). B, The top and middle panels are immunolocalization of PDE5 in rat urinary bladder. Representative double immunofluorescence staining for sham (top) and PBOO rat (middle) bladder was conducted. DAPI (blue fluorescence), α‐SMA (green fluorescence), PDE5 (red fluorescence) and merged images are shown from left to right (magnification × 200). The bottom panels were positive control for PDE5 using rat lung tissue (magnification × 400) and negative control by omitting the primary antibody (magnification × 200)

We further carried out a confocal microscopy study (Figure 6A,B). Both for human and rat, the PDE5 exhibited almost entirely in the SM bundles, and this co‐immunolocalized with α‐SMA. Both positive and negative controls reacted as expected.

### 3.6 Expression of molecules related to SM contraction and relaxation pathways are altered in human and rat PBOO bladder

**Figure 7:**
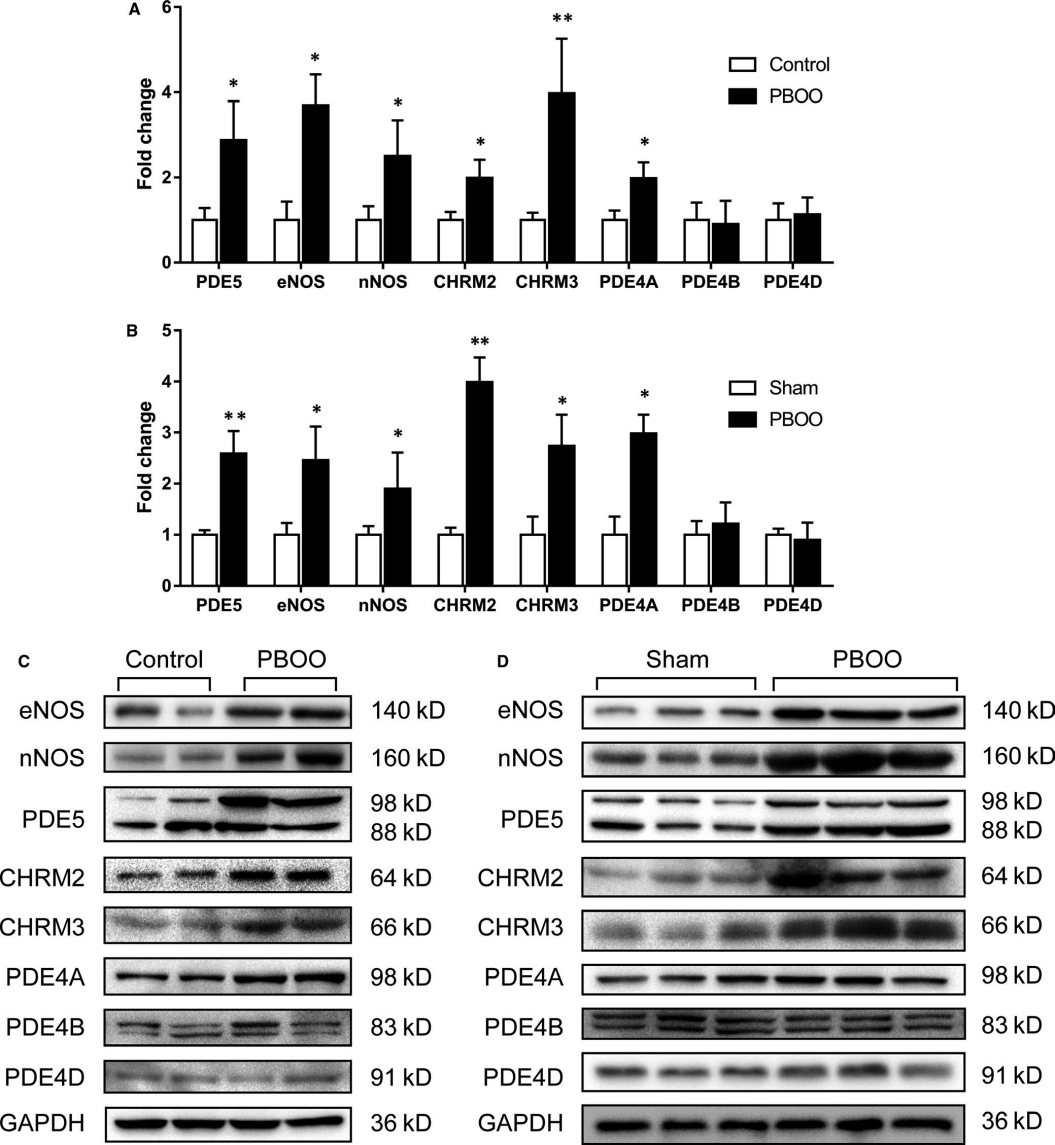
Expression of NOS isoforms, PDE5, CHRM isoforms and PDE4 isoforms in human and rat urinary bladders. A, mRNA expression level of target genes in human bladder. White column, control; black column, PBOO. *P < 0.05 vs control; **P < 0.01 vs control. B, mRNA expression level of target genes in rat bladder. White column, sham; black column, PBOO. *P < 0.05 vs sham; **P < 0.01 vs sham. Data are expressed as mean ± SEM. Experiments were repeated three times for each sample (n = 10 for each group). C, Representative bands of various proteins expressed in human bladder. The two on the left are control group and the other are PBOO group. D, Typical bands of various proteins expressed in rat bladder. The three on the left are sham group and the other are PBOO group. Molecular weight (kD) is indicated to the right of the blot. The GAPDH was blotted and used as a loading control

The expression of important molecules in the NO/PDE5 pathway, CHRM isoforms and PDE4 isoforms of human and rat bladder were determined by RT‐PCR (Figure 7A,B) and Western blotting (Figure 7C,D). For both human and rat, eNOS, nNOS, PDE5, CHRM2, CHRM3 and PDE4A were increased in the PBOO group, whereas PDE4B and PDE4D were not changed, either at mRNA or at protein level.

## 4 DISCUSSION

The current study demonstrates for the first time that PDE5 expression is up‐regulated in the urinary bladders of PBOO rats and human. Our novel data also show alterations of SM contractility and related molecules in PBOO bladders. Our study suggests that the up‐regulation of PDE5 in obstructed bladder could contribute to the non‐changed Q_max_ for BPH/LUST patients treated with PDE5is.

To our knowledge, obstruction has a significant impact on the bladder's structure and physiology. When obstruction emerges, compensatory changes in the bladder wall occur to generate additional pressure and overcome the rising resistance from the outlet. In this ‘compensated stage’, bladder SM becomes hypertrophy and hyperplasia with an increased bladder wall thickness to maintain voiding.^25^, ^26^ With the outlet resistance continuously rising, increased connective tissue deposition and fibrosis, along with attenuated detrusor contraction, were exhibited, and the bladder enters in ‘decompensated stage’.^25^, ^27^ Indeed, our present study found 2‐week partial obstruction of the rat bladder resulted in increased bladder weight and SM hypertrophy without obvious fibrosis. Different from the rat model, PBOO patients showed not only an elevated SM component but also obvious fibrosis. To further determine whether the pathological conditions of SM area increase resulted from hyperplasia and/or hypertrophy, we found that SM cells from both human and rat obstructed bladder increased in size (diameter) but not in numbers, based upon which we speculated that the pathological changes in obstructed bladder were mainly SM hypertrophy rather than hyperplasia.

Our study also found that the contractility of PBOO bladder strips was heightened to carbachol stimulation both for rats and human. It is well known that M_2_ and M_3_ receptors predominantly modulated the contraction of bladder SM and their dysregulation may lead to change in bladder SM contractility. In line with a previous study in a PBOO rat model^28^, ^29^ and BOO patients,^30^ our molecular biology experiments demonstrated that M_2_ and M_3_ receptors were both up‐regulated (at both the mRNA and protein levels) in the PBOO groups, which could be responsible for the increased CC‐induced contraction. Also, carbachol EC50 values were different between PBOO and control (or sham) groups (in Figure 4A,B). There are at least five subtypes of muscarinic receptors that exist in the mammalian bladder (M1‐M5), and our current study found the up‐regulation of M2 and M3 receptors (in Figure 7) with no change for other subtypes (M1, M4 and M5, data not shown). Therefore, the difference in carbachol EC50 may be caused by alteration of M2 and M3 expression in the bladder wall. Meanwhile, our Masson's trichrome staining study observed the percentage of SM was increased both in rat and human PBOO group, which could also lead to the higher responsiveness to carbachol muscarinic stimulation for bladder tissues. Additionally, we found that bladder strips from PBOO rats showed an elevated shortening velocity and poor maintenance of force, which was not observed for human bladder. Different SM myosin isoform compositions could contribute to the different shortening velocity between rats and human.^31^ However, further studies are required.

Contrary to our findings, a recent study from Silva‐Ramos et al^32^ have demonstrated that acetylcholine‐induced contractions attenuated in detrusor samples from human patients with BPH. Since the bladders could have entered into a ‘compensated stage’ or ‘decompensated stage’ after obstruction, the contraction force would change accordingly. Considering that the time spent from ‘compensated stage’ to ‘uncompensated stage’ was different for individuals, we speculated that the difference between the two studies might be attributed to the PBOO bladders being obtained in different stages of compensation/decompensation. Moreover, urothelium‐denuded detrusor samples were used in Silva‐Ramos's study, which was different from our study. Muscarinic receptors were found on the urothelium at high density; thus, activation of the muscarinic receptors in the urothelium releases substances that modulate afferent nerves and SM activity. Therefore, the existence of urothelium or not may cause a difference in bladder contraction.

Importantly, our current study demonstrated that PDE5 was up‐regulated in obstructed bladder from both humans and rats. Similar to previous reports,^33^, ^34^ PDE5 was almost entirely immunolocalized in the SM bundles. In the past decades, modulation of PDE5 in corpus cavernosum (CC) was widely explored but there were few studies on bladder PDE5. Previous studies did, however, suggest that endothelial NOS (eNOS) and exogenous NO donors can modulate PDE5 expression in mice CC.^35^, ^36^ In our study, eNOS and nNOS were both up‐regulated in the PBOO group. Therefore, we speculate that NOS might modulate PDE5 expression in bladder. In general, eNOS was expressed in vascular endothelium and urothelium,^37^ whereas nNOS was expressed in neurons or nerves.^38^ It is suggested that histological changes originating from obstruction may play a role in regulation of NOS expression. When obstructed bladder entered the compensatory stage, the bladder wall thickened and nerve growth factor (NGF) was increased,^39^ which might result in more endothelial cells and nerve fibres than normal bladder. In the present study, PBOO bladders both from rat and human are thought to be in compensatory stages due to exaggerated detrusor contraction induced by a muscarinic receptor agonist. Consistently, several other studies demonstrated that both eNOS and nNOS were more highly expressed in BOO rat bladder.^40^, ^41^ Meanwhile, as PDE5 was mostly localized in bladder SM, the increase of SM observed in the present study could also contribute to the PDE5 up‐regulation. However, although the expressions of PDE5 were altered in human and rat bladder tissues, it was not known exactly in which cell type this change occurred. Thus, separation of different cell types and then performing single cell PCR could indeed be an important and interesting future study. To further explore whether the PDE5 activity changed in the PBOO group, we conducted the in vitro relaxation experiment. As our present data showed NOS expression was changed in PBOO the group, which may mediate NO formation and downstream PDE5 in bladder, we used SNP (NO donor) to eliminate the effect of NO formation. In line with previous studies,^33^, ^34^ our present study showed that increasing concentration of SNP induced a dose‐dependent relaxation effect on bladder strips, and the relaxation was enhanced by vardenafil. Considering the concentration (100 nmol/L) of vardenafil used in our study only caused a weak relaxation for CC pre‐contracted bladder strips (less than 10%), we concluded that the enhanced effect on SNP‐induced relaxation (Figure 5) was not due to the effect of Vardenafil itself. Nevertheless, the relaxant responsiveness to SNP for PBOO bladder was weaker than that in control. Despite this, SNP could still significantly relax PBOO preparations when compared with vehicles (Figure S1). However, this relaxation effect was augmented to the same level as the control group when vardenafil was pre‐administered. The PDE5 up‐regulation in PBOO bladder could contribute to less responsiveness to SNP but exaggerated PDE5is effect. Consistent with a previous castration rat model,^42^, ^43^ CC and prostate strips showed greater responsiveness to SNP but worse responsiveness to PDE5 inhibitor probably due to the PDE5 reduction after bilateral orchiectomy.

A number of studies have demonstrated the in vitro relaxant effect of PDE5is on bladder neck strips and a recent review^16^ stated that the PDE5is relaxant effect is more evident in bladder neck preparations with an NO donor (SNP) used, which suggested that endogenous NO plays little role in PDE5is relaxant effect in vitro than an exogenous NO donor. Moreover, a previous study^14^ found that sildenafil‐induced relaxation of human detrusor SM involved cGMP‐, cAMP‐ and K + channel‐dependent signalling pathways, although its relaxant effect was only obvious in higher sildenafil concentration. Meanwhile, PDE5is may also relax bladder SM through regulating the RhoA/ROCK pathway.^16^ It is believed that the relaxant effect of vardenafil was related to PDE5 protein expression, but its accurate mechanism remains undefined and needs further exploration.

Moreover, it should be taken into consideration that full‐thickness bladder preparations including urothelium and lamina propria were used in our current study when interpreting the data. Besides the primary role of the urothelium to form a relatively impermeable barrier to protect underlying tissue, more evidence has demonstrated that urothelium could modulate afferent signalling and detrusor SM activity such as through muscarinic receptors and NO release.^44^ In addition, the lamina propria has been theorized to be the ‘functional’ centre for co‐ordinating the activities of the urothelium and detrusor SM.^45^ Therefore, it has been suggested that the functional result of a drug might be through not only a direct action on the detrusor but also indirectly via a possible mechanism involving the urothelium and lamina propria. However, our study showed PDE5 was mainly localized in SM bundles and blood vessels of bladder, whereas a previous study also showed the same result and did not detect the PDE5 expression in urothelium.^33^ This suggested that the level of functional activity of PDE5 might not be affected by the existence of urothelium. However, blood vessels could be the site of action of PDE5i, hence exhibiting relaxation response to improve bladder blood flow, which may have influence on our result.

In addition to the NO/cGMP‐PDE5 axis, the intracellular cAMP‐PDE4 pathway could also relax the SM and mediate the phasic contraction of bladder.^46^ PDE4 is cAMP‐specific^47^ and it has four isoforms that include PDE4A, PDE4B, PDE4C and PDE4D whereas PDE4C was found not to be expressed in rat bladder.^48^ Inhibition of PDE4 has a moderate relaxation effect on detrusor strips of human,^49^ rabbit^50^ and guinea pig,^51^ but a strong relaxation effect on bladder neck of human and pig.^52^ In BOO rats, PDE4 inhibitors decreased the frequency and amplitude of non‐voiding contractions and suppressed bladder overactivity.^53^ Moreover, PDE4 inhibitors also showed an anti‐inflammatory effect and improved bladder function in diabetic bladder,^54^ obesity‐associated OAB^55^ and experimental cystitis.^56^, ^57^ In our current study, we demonstrated that the PDE4A isoform was upregulated with no change in PDE4B and PDE4D in the PBOO group for both rat and human. However, whether PDE4 could be a new therapeutic target for bladder disease remains controversial and in need of further investigations.

In our current study, age difference between BPH patients (70.38 ± 6.05 years) and control individuals (31.54 ± 6.75 years) may have potentially influenced the results, as previous studies have demonstrated that ageing seems to induce hypo‐function and histological changes of the bladder and urethra in humans. Therefore, it is necessary to perform an age‐matched cohort study. However, it is well known that elderly men often suffered BPH/LUTS which increases with age with incidence reaching 90% for men over 85 years old. Thus, it was hard for us to find aged men without bladder outlet obstruction as controls. Actually, a recent study from Silva‐Ramos et al^32^ demonstrated that ageing did not seem to influence the sensitivity of detrusor SM to cholinergic stimulus in obstructed bladders using correlation analysis. In addition, our age‐matched rat study showed consistent results with non–age‐controlled human experiments. But it will be intriguing to conduct further study using an age‐matched cohort in the future.

In summary, obstructed bladder showed significant changes in histology and physiology, as well as related contractility molecules. The up‐regulation of PDE5 could contribute to the lack of effect on Q_max_ for BPH/LUTS patients treated with PDE5is. However, PDE5 (with presence of NO) and PDE4 may be new therapeutic targets for bladder diseases.

## CONFLICT OF INTEREST

No potential conflict of interest was reported by the authors.

## AUTHOR CONTRIBUTIONS

**Weixiang He:** Conceptualization (equal); Data curation (equal); Investigation (equal); Methodology (equal); Validation (equal); Visualization (equal); Writing‐original draft (equal); Writing‐review & editing (equal). **Han Xiang:** Conceptualization (equal); Data curation (equal); Investigation (equal); Methodology (equal); Validation (equal); Visualization (equal). **Daoquan Liu:** Investigation (equal); Methodology (equal); Validation (equal). **Jianmin Liu:** Investigation (equal); Methodology (equal); Validation (equal). **Mingzhou Li:** Formal analysis (equal); Software (equal). **Qian Wang:** Formal analysis (equal); Software (equal). **Qiaofeng Qian:** Resources (equal); Software (equal). **Yan Li:** Resources (equal); Validation (equal). **Xun Fu:** Formal analysis (equal); Resources (equal). **Ping Chen:** Formal analysis (equal); Methodology (equal). **Yuming Guo:** Methodology (equal); Software (equal). **Guang Zeng:** Resources (equal); Validation (equal). **Zhonghua Wu:** Methodology (equal); Resources (equal). **Daxing Zhan:** Methodology (equal); Resources (equal). **Xinghuan Wang:** Methodology (equal); Software (equal). **Michael E DiSanto:** Methodology (equal); Writing‐review & editing (equal). **Xinhua Zhang:** Conceptualization (equal); Data curation (equal); Funding acquisition (equal); Methodology (equal); Project administration (equal); Resources (equal); Software (equal); Supervision (equal); Visualization (equal); Writing‐review & editing (equal).

## Supporting information

Fig S1

Fig S2

Table S1

## Data Availability

The data used to support the findings of this study are available from the corresponding author upon request.
