# Supplementary figures and images for: Changes in the expression and function of the PDE5 pathway in the obstructed urinary bladder
Source: J Cell Mol Med. 2020 Oct 3;24(22):13181–95. doi: 10.1111/jcmm.15926 (PMC7701571; doi:10.1111/jcmm.15926)

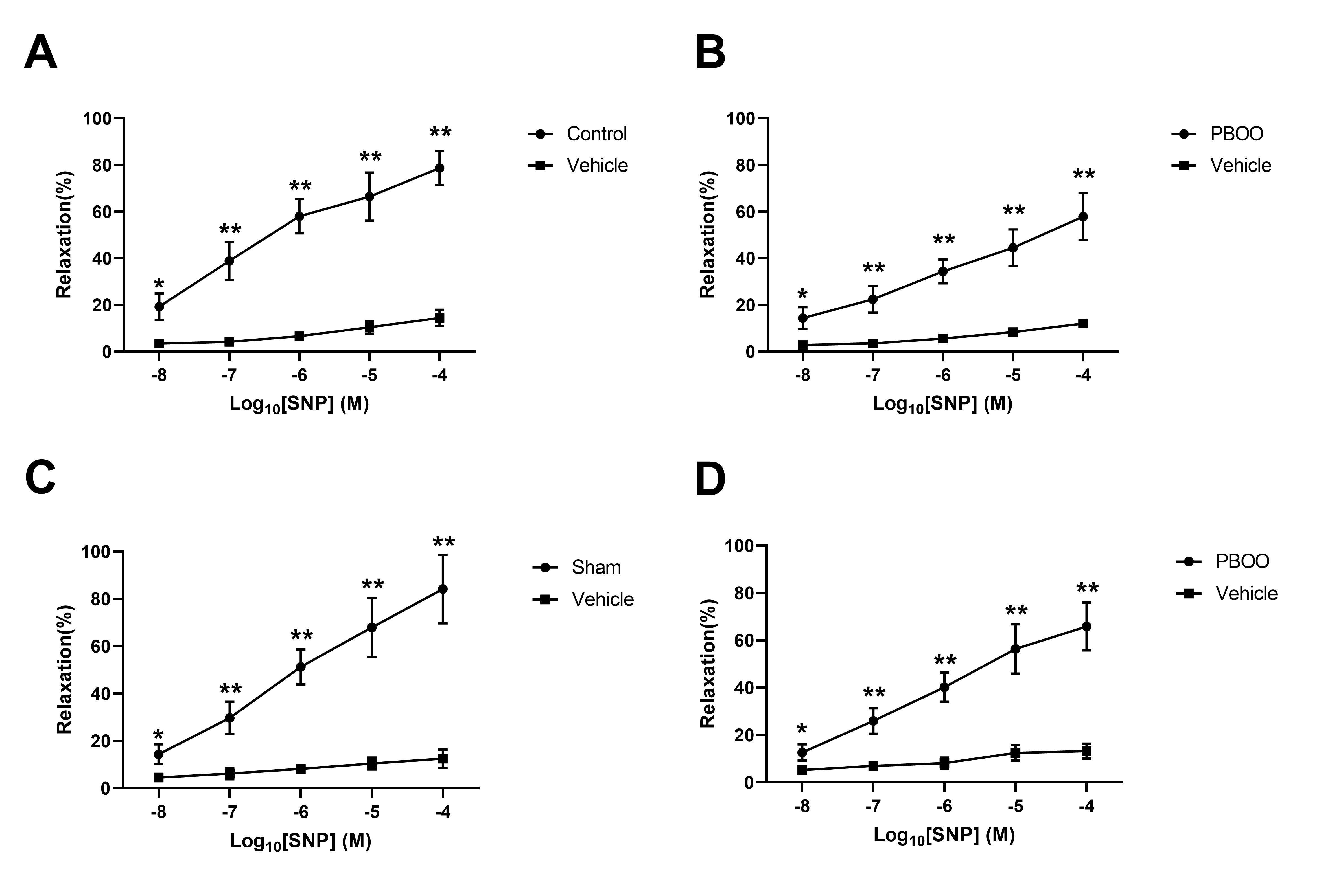

Supplement: Supplementary file 1 — Fig S1 [file JCMM-24-13181-s001.tif]

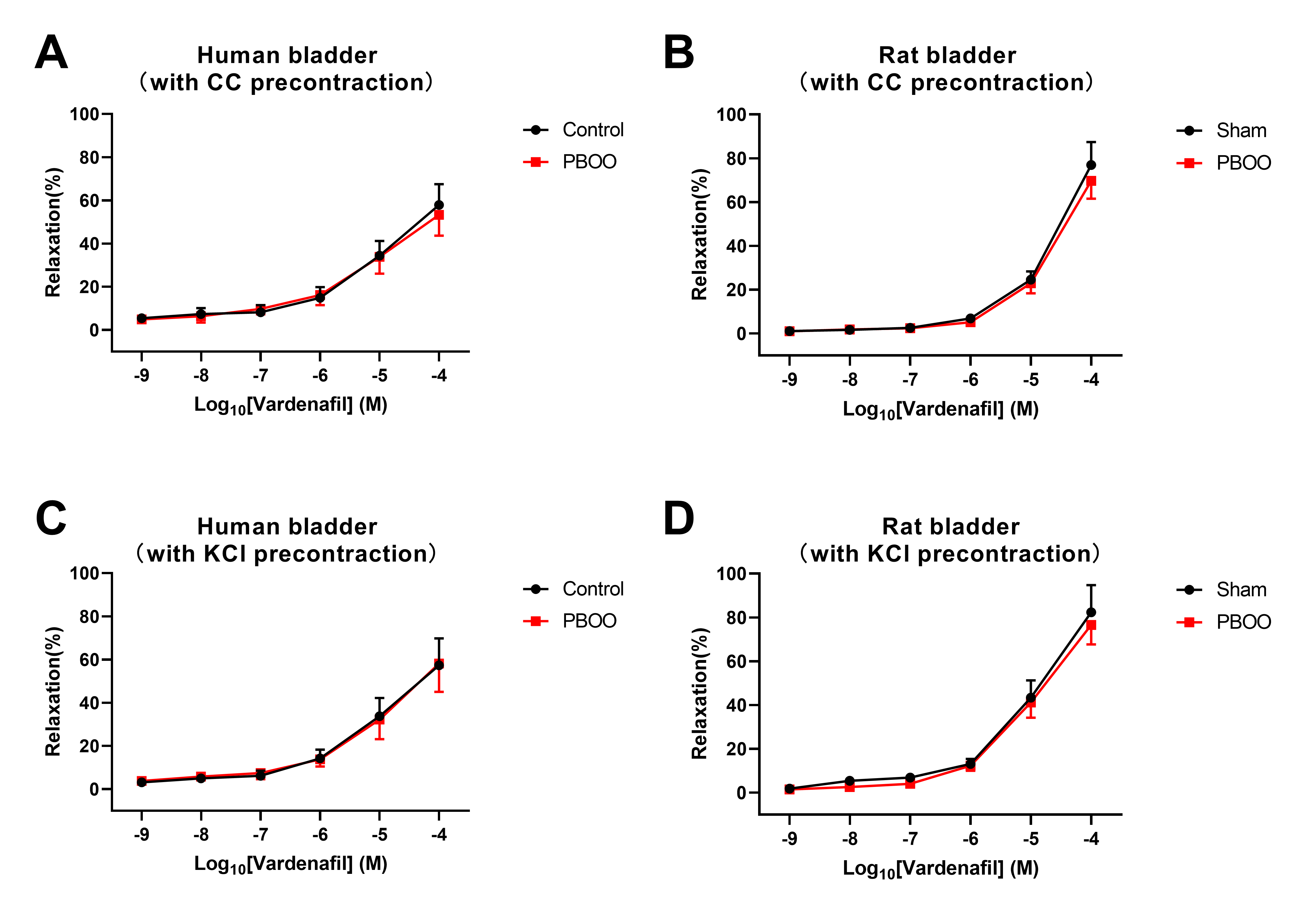

Supplement: Supplementary file 2 — Fig S2 [file JCMM-24-13181-s002.tif]
