# Supplementary material for: Changes in the expression and function of the PDE5 pathway in the obstructed urinary bladder
Source: J Cell Mol Med. 2020 Oct 3;24(22):13181–95. doi: 10.1111/jcmm.15926 (PMC7701571; doi:10.1111/jcmm.15926)
Supplement: Supplementary file 3 — Table S1 [file JCMM-24-13181-s003.docx]

| Group | SM cell diameter (μm) | | |  | SM cell numbers | | |
| --- | --- | --- | --- | --- | --- | --- | --- |
|  | Mean | Minimum | Max |  | Mean | Minimum | Max |
| Rat | | | | | | | |
| Sham (n=10) | 4.15 ± 0.37 | 2.98 ± 0.27 | 5.69 ± 0.43 |  | 389.2 ± 29.1 | 267.7 ± 31.1 | 489.1 ± 41.8 |
| PBOO (n=10) | 5.32 ± 0.65* | 3.34 ± 0.45* | 6.56 ± 0.71* |  | 375.4 ± 47.3 | 293.5 ± 37.3 | 541.3 ± 60.4 |
| Human | | | | | | | |
| Control (n=10) | 4.87 ± 0.45 | 3.82 ± 0.27 | 6.23 ± 0.71 |  | 537.6 ± 37.2 | 327.2 ± 38.9 | 757.1 ± 65.3 |
| PBOO (n=10) | 5.89 ± 0.52^#^ | 4.61 ± 0.33^#^ | 7.17 ± 0.66^#^ |  | 552.1 ± 56.7 | 356.2 ± 43.8 | 734.1 ± 89.6 |

**Supplementary Table 1. Analysis of diameter and numbers of detrusor SM cells in Masson’s trichrome staining micrographs (25x).**

*p<0.05 vs. Sham; ^#^p<0.05 vs. Control;

Data are expressed as mean±SD.
